# Pheochromocytoma/Paraganglioma (PPGL): A Misdiagnosed Cause of Hypertension during Pregnancy

**DOI:** 10.1155/2024/6655229

**Published:** 2024-03-27

**Authors:** Rafael Buck Giorgi, Priscila Teixeira Aroucha, Thalissa M. Favreto, Micaela F. Montero, Julia M. F. Velloni, Ilana Korkes, Elisa Napolitano Ferreira, Caroline Olivati, Jose Viana Lima, Claudio E. Kater, Flavia A. Costa-Barbosa

**Affiliations:** ^1^Adrenal and Hypertension Unit, Division of Endocrinology and Metabolism, Department of Medicine, Federal University of Sao Paulo Medical School-EPM/Unifesp, São Paulo, Brazil; ^2^Division of Endocrinology, Faculty of Medical Sciences and Health, Pontific Catholic University of São Paulo, Sorocaba, Brazil; ^3^Research and Development Division, Fleury Group, São Paulo, Brazil; ^4^Division of Endocrinology and Metabolism, Department of Medicine, Santa Casa de São Paulo, São Paulo, Brazil

## Abstract

Hypertension (HT) during pregnancy is not an infrequent obstetric problem, reaching a prevalence of 5-10%. This condition is highly associated with both maternal and fetal complications if not precisely diagnosed and managed. Even though primary HT, obesity, and preeclampsia are the main causes of HT in this period, other less familiar conditions must be considered during the investigation. Pheochromocytoma and paraganglioma (PPGL) are chromaffin cell tumors that produce, store, and secrete catecholamines, leading to HT and other adrenergic manifestations. Recognition of PPGL is crucial since misdiagnosis and improper management can lead to high morbidity and mortality, particularly during pregnancy. We report on two cases of PPGL diagnosed during pregnancy with different managements. Case 1 is a 25-year-old female at 31 weeks of first pregnancy, whose severe HT and life-threatening symptoms prompted an emergency delivery without previous confirmation or medical treatment of a suspected PPGL. After confirmation, a right adrenal PPGL was surgically resected 4 months later, following 15 days of medical therapy. Case 2 is a 22-year-old female at 18 weeks of pregnancy whose symptomatic PPGL was resected in the second trimester. A next-generation sequencing panel, including 23 PPGL-related genes, found no germline pathogenic variants (GPVs) in case 1 and an exon 1-4 germinative heterozygous deletion of the *MAX* gene in case 2. Despite the different medical approaches, both cases had satisfactory outcomes. Although uncommon, PPGL should be considered in the differential diagnosis of HT in pregnancy since missing the diagnosis and failing to introduce appropriate and timely treatment may lead to dramatic consequences for the mother and fetus. PPGL diagnosed during reproductive age is likely to result from GPV, prompting genetic investigation and counseling.

## 1. Introduction

Pheochromocytoma/paraganglioma (PPGL), diagnosed during pregnancy, is considered a rare event with an underestimated prevalence of around 7 : 100,000 pregnancies [1]. PPGL are neuroendocrine tumors arising from the adrenal medulla (pheochromocytoma) or extra-adrenal paraganglia (paraganglioma), characterized by catecholamine synthesis, release, and metabolism [2, 3]. In a series of pregnancies followed over 22 years, the diagnosis of pheochromocytomas predominated over those of paragangliomas: 89% × 11% [4]. The adrenergic consequences of PPGLs are hypertension (HT), diabetes mellitus, and other common symptoms during pregnancy, contributing to increased maternal and fetal morbimortality. In recent systematic reviews, including 135 PPGL cases diagnosed during pregnancy, maternal and fetal mortality rates were 8% and 17%, respectively [4], much less than previous misdiagnosed reports before 1970 (48% and 54%, respectively), but yet with significant morbidity [5]. Several PPGL features, such as location, size, and biochemical pattern, may influence the maternal/fetal prognosis [6]. During the next-generation sequencing (NGS) era, complementary genetic aspects have added newer insights into treating patients with PPGL, even during pregnancy [4, 7]. Here, we report the phenotype and genotype data of two patients with PPGL diagnosed during pregnancy and review the literature on management and pitfalls that can occur during this period.

## 2. Case Reports

### 2.1. Case 1

A 25-year-old female was admitted to the Obstetrician-Gynecologist (ObGyn) Service of the University Hospital at 31 weeks of her first pregnancy due to an episode of HT during a dental procedure. She had been allegedly healthy before pregnancy. On admission, the physical exam was remarkable for a supine high blood pressure (BP) of 150 × 90 mmHg, heart rate (HR) of 130 bpm, and no lower limb edema. Pregestational body mass index (BMI) was 18 kg/m^2^. Blood analysis revealed mild anemia, leukocytosis without deviation, and altered inflammatory tests. Then, a presumptive preeclampsia diagnosis was made. However, BP became difficult to control, and hyperglycemia appeared. Detailed medical history disclosed the prior sweating and palpitations that persisted during gestation. PPGL was suspected, and plasma metanephrines were requested.

**Figure 1:**
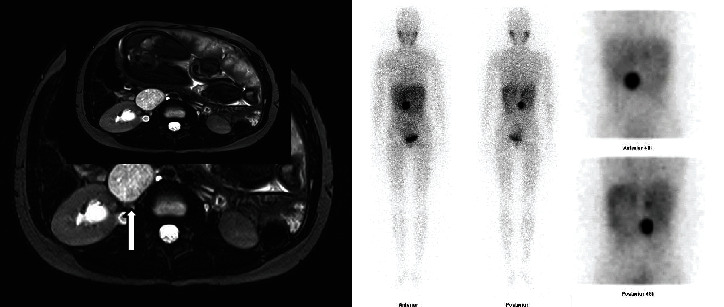
Case 1. (a) Abdominal MRI showing a 41 × 33 × 42 mm retroperitoneal mass with T2 enhancement near the right kidney. (b) Postpartum [^131^I]-metaiodobenzylguanidine (MIBG), showing basal and post-48-hour uptake (anterior and posterior) compatible with a right kidney paramedian paraganglioma.

An abdominal magnetic resonance imaging (MRI) revealed a 41 × 33 × 42 mm retroperitoneal mass near the right kidney with T2 enhancement, consistent with paraganglioma (Figure 1(a)). Because the patient persisted with labile HT, the ObGyn staff decided to terminate the pregnancy at 32 weeks. An emergency cesarean section (C-section) was performed, with no previous adrenergic receptor blockade. During surgery, BP reached 260 × 192 mmHg and HR 225 bpm. The patient required norepinephrine immediately after delivery, followed by sodium nitroprusside during the first postoperative period. Soon after, she was started on oral alpha-blockers, which contraindicated breastfeeding. The newborn weighed 2370 grams and had Apgar scores of 8 and 9 at 1 and 5 minutes, respectively. The placenta weighed 430 grams and had three blood vessels, a 3rd trimester morphology, bleeding, and focal villous necrosis, with no histological changes in the membrane or umbilical cord. One week after delivery, plasma normetanephrines were 10-fold the upper limit of normal (ULN), and metanephrines were normal. Iodine-131 metaiodobenzylguanidine (^131^I-MIBG) scintigraphy (whole-body scintigraphy to screen paraganglioma) [2] disclosed evident radioiodine uptake above the right kidney, confirming PPGL (Figure 1(b)). Video laparoscopic mass resection was performed four months after delivery, following successful adrenergic blockade. Pathologic examination revealed a 19 g PPGL (4.0 × 3.4 × 2.6 cm ovoid mass) with a low histological grade. BP and plasma glucose have normalized postoperatively, and outpatient follow-up was conducted without complications.

### 2.2. Case 2

A 22-year-old female was admitted to the ObGyn Service at the University Hospital on the 18th week of gestation with episodic HT. Her supine BP was 180 × 110 mmHg and HR 95 bpm, despite the use of methyldopa (500 mg three times daily); orthostatic hypotension was present. BMI was 29.8 kg/m^2^, and the fetus was in good condition. Abdominal ultrasound identified a right adrenal mass of approximately 8 cm in diameter, suggesting pheochromocytoma and prompting antihypertensive therapy to be replaced with prazosin. Urinary normetanephrine was nine times higher than the ULN with normal metanephrines. Abdominal MRI disclosed a large mass close to the right adrenal with no T2 enhancement. Prazosin therapy required for BP control reached 12 mg/day, and a beta-blocker was introduced to control tachycardia. Video laparoscopic resection of the tumor and the right adrenal was performed at 23 weeks, 15 days after alpha-blockade. Normal BP followed the procedure, and the patient did not require vasoactive drugs in the intensive care unit. Pathologic examination was compatible with PPGL. The pregnancy was terminated at 39 weeks by vaginal delivery with no incidents. The newborn weighed 2370 grams and had Apgar scores of 9 and 10 at 1 and 5 minutes, respectively. The patient remained currently normotensive and asymptomatic.  Table 1 summarizes clinical data from both patients.

### 2.3. Molecular Study

DNA was extracted from peripheral blood leukocytes using QIAsymphony DNA Mini Kit®, according to manufacturer recommendations. The DNA library was prepared using a custom-captured-based target enrichment kit. The custom panel was designed to allow sequence to the complete coding regions and flanking splice sites and copy number variations (CNV) of 23 genes related to PPGL: *ATM*, *ATR*, *CDKN2A*, *EGLN1*, *FH*, *HRAS*, *KIF1B*, *KMT2D*, *MAX*, *MDH2*, *MERTK*, *MET*, *NF1*, *PIK3CA*, *RET*, *SDHA*, *SDHAF2*, *SDHB*, *SDHC*, *SDHD*, *TMEM127*, *TP53* (including promoter region), and *VHL*. Sequencing was performed using an Illumina NextSeq platform. Careful analysis was performed using custom bioinformatic tools for reading mapping and variant calling and the SOPHiA DDM® platform for variation annotation and interpretation.

In case 1, no GPV has been detected in any of the 23 genes studied, whereas a pathogenic exon 1-4 germinative heterozygous deletion in the *MAX* gene was identified in case 2.

## 3. Discussion

We reported two pregnant women harboring PPGLs who have been managed differently and yet attained favorable maternal and fetal outcomes (Table 1). PPGL is misdiagnosed in almost 30% of hypertensive pregnancies and is frequently recognized after an acute or severe complication [8], as described in case 1. Accordingly, the actual prevalence of PPGL should be higher than reported [9].

PPGL symptoms are often indistinguishable from other hypertensive diseases in pregnancy (Table 2). Unlike preeclampsia, PPGL-associated HT usually starts at early gestational age with no lower limb edema or proteinuria. Other PPGL symptoms may include paroxysmal HT, orthostatic hypotension, dizziness, palpitations, or hyperglycemia [10–12]. Moreover, typical PPGL spells can also be precipitated by anesthesia, uterine contractions, vigorous fetal movements, mechanical compression by the gravid uterus, or antiemetic agents. Although the placenta retains an efficient enzymatic apparatus that restrains maternal catecholamines, these vasoactive compounds lead to vasoconstriction of the uteroplacental circulation, causing intrauterine fetal insufficiency, fetal hypoxia, spontaneous abortion, and fetal death [5, 12].

Recently, Wing et al. have shown better maternal and fetal outcomes in paragangliomas than pheochromocytomas, with lower maternal and fetal mortality rates. Moreover, pregnant women with paragangliomas are more asymptomatic than those with pheochromocytomas (4.5% vs. 27%) and have fewer hypertensive crises (14% vs. 27%) [7]. A comprehensive systematic review involving 249 pregnancies in 232 patients with PPGL verified that unrecognized and untreated PPGLs during pregnancy are positively associated with maternal or fetal death and adverse maternal outcomes [6]. In addition, the presence of an abdominal or pelvic tumor (leading to uterus compression and additional catecholamine stimulation) and marked increased levels of catecholamines (10 times the ULN) were also associated with unfavorable outcomes [6]. Therefore, when a PPGL is suspected, measurements of plasma or urinary metanephrines are the preferred tests for screening [2]. Once the biochemical diagnosis is confirmed, imaging procedures are necessary for tumor location. MRI is highly recommended in pregnancy [7, 10]. The other traditional imaging techniques, such as computed tomography, radionuclide imaging (e.g., [^123^I] and [^131^I]-MIBG), and more recently positron emission tomography using dopamine, glucose, or somatostatin analogues (^18^F-DOPA, ^18^F-FDG, or ^68^gallium) [13], are contraindicated during pregnancy due to the associated risk of fetal exposure to ionizing radiation [5].

Once a PPGL is identified, surgery is indicated. Available studies suggest that laparoscopic surgery should be performed before 24 weeks gestation or delayed until the time or after delivery [5, 14]. However, Bancos et al. have shown that surgery during pregnancy was not associated with a better prognosis [6]. Then, a multidisciplinary team involving endocrinologists, ObGyn, surgeons, and anesthesiologists, all prepared for the drawbacks of PPGL, is essential to care for such patients [5, 14]. If surgery cannot be performed during pregnancy, tumor removal concomitant with a C-section (preferred delivery method) or postpartum operation is the appropriate procedure [14]. Pharmacological therapy is associated with successful outcomes [6]. Alpha-adrenergic blockade associated with increased salt and fluid intake is essential to reduce the risk of intraoperative and perioperative complications, and it should be started as early as possible after diagnosis [6]. Doxazosin, a selective alpha-1 adrenergic receptor blocker, is a rational choice, and the goal is to achieve a low normal systolic blood pressure [15]. Care must be paid to address the potential risk of postsurgical hypotension in mothers and hypotension and respiratory depression in newborns of mothers treated with phenoxybenzamine [16]. If tachyarrhythmia is a concern, beta-blockers may be introduced with caution at least seven days after alpha-blockade since they may cause intrauterine fetal growth insufficiency [4, 10]. Preference is given to cardioselective beta-1 blockers (such as metoprolol succinate) as beta-2 receptor blockade in the myometrial layer of the uterus could reduce myometrium relaxation [15].

In a retrospective study of pregnancies with PPGL, 66% of the patients carried GPVs in an autosomal dominant fashion [6]. So far, more than 26 genes have been associated with PPGL [17, 18]. In case 2, an exon 1-4 heterozygous deletion in the *MAX* gene has been detected. *MAX* is part of the PPGL group of tumor susceptibility genes (cluster 2: *NF1*, *RET*, and *TMEM127*) involved in activating kinase signaling pathways. *MAX* acts as a partner of proto-oncogene *MYC* [19] and is involved in cell proliferation, differentiation, and apoptosis [20]. Comino-Méndez et al. have also described *MAX* as a tumor suppressor gene [21]. This gene GPV has been associated with a predisposition to PPGL in about 1% of inherited PPGL syndromes [22]. Pathogenic variants may result from loss of heterozygosity of the *MAX* allele due to paternal uniparental disomy and loss of the maternal allele [21]. Bilateral or unilateral diseases have been reported with a variable risk of malignancy [22, 23]. Case 2 had increased normetanephrines and normal metanephrines, a phenotypic feature of *MAX* individuals [22]. Some cases of PPGL carrying *MAX* GPV or deletion may also present pituitary adenomas with a variable secretory pattern [24, 25]. Although no pituitary imaging has been performed in our case, spontaneous pregnancy suggests a favorable hypothalamic-pituitary environment.

In summary, although uncommon, PPGL should be considered in the differential diagnosis of HT in pregnancy, especially if HT has an early onset or worsens during gestation. Inappropriate diagnosis and treatment may lead to dramatic consequences for the mother and fetus, such as fetal hypoxemia, intrauterine growth restriction, prematurity, and fetal death. PPGLs diagnosed during reproductive age most likely result from a GPV, prompting a genetic investigation. If positive, genetic counseling is imperative, as first-degree relatives of individuals carrying pathogenic PPGL variants have a 50% chance of being carriers. Thus, early diagnosis of PPGL is critical to ensure a better maternal-fetal outcome, allowing adequate surgical preparation and a correct delivery decision to reduce morbimortality. Although a pregnancy diagnosis of PPGL could be a terrible moment for the mother, secure multidisciplinary team management can give relative relief to the future family.

## Figures and Tables

**Table 1:** Clinical data from the two patients diagnosed with PPGL during pregnancy.

	Case 1	Case 2
Age at diagnosis	25 years	22 years
Gestational age	31 weeks	18 weeks
Clinical history and symptoms	Hypertension, hyperglycemia, sweating, palpitations since before pregnancy	Hypertension during paroxysms
Plasma NMN/MN	10x ULN/normal	—
Urinary NMN/MN	—	10x ULN/normal
Molecular study	None GPV detected	*MAX* deletion exon 1-3
Surgical management	C-section at 32^nd^ week, no previous adrenergic blockade; laparoscopic excision 4 mo later	Laparoscopic tumor excision at 23 wks, 15 days after alpha-adrenergic blockade
Maternal and fetal complications	Hypertensive crisis during C-section; no further clinical repercussions	None

NMN = normetanephrine; MN = metanephrine; ULN = upper limit of normal.

**Table 2:** Clinical features most likely related with pregnancy-related hypertension or pheochromocytoma/paraganglioma.

Pregnancy-related hypertension	Pheochromocytoma/paraganglioma
Hypertension > 26 wks	Hypertension < 20 wks
	Paroxysmal hypertension
	Orthostatic hypotension
Syndromic features	Syndromic features
Nausea	Nausea
Edema and proteinuria	Paroxysmal headache
HELLP syndrome	

## Data Availability

The data that support the findings of this study are available from the corresponding author, FACB, upon request.

## Abbreviations

^131^I-MIBG: Iodine-131 metaiodobenzylguanidine
BMI: Body mass index
BP: Blood pressure
C-section: Cesarean section
CNV: Copy number variations
GPV: Germline pathogenic variants
HR: Heart rate
HT: Hypertension
MRI: Magnetic resonance imaging
NGS: Next-generation sequencing panel
ObGyn: Obstetrician-gynecologist
PPGL: Pheochromocytoma and paraganglioma
ULN: Upper limit of normal.

## Additional Points

*Established Facts and Novel Insights*. The established facts are as follows. (i) Pheochromocytoma/paraganglioma (PPGL) in pregnant women is a rare, but life-threatening condition, especially if not properly identified and managed. (ii) Thus, PPGL and other secondary causes of hypertension should be investigated in unusual presentations of hypertension during pregnancy. The novel insights are as follows. (i) These case reports exemplify that closer follow-up will increase the chances of good maternal/fetal outcomes if PPGL is diagnosed during gestation, despite different clinical contexts. (ii) PPGL diagnosed during the reproductive age is likely to result from a germline pathogenic variant and must be genetically investigated. Once positive, genetic counseling is imperative.
